# Assessment of Pb(II), Cd(II), and Al(III) Removal Capacity of Bacteria from Food and Gut Ecological Niches: Insights into Biodiversity to Limit Intestinal Biodisponibility of Toxic Metals

**DOI:** 10.3390/microorganisms9020456

**Published:** 2021-02-22

**Authors:** Fanny George, Séverine Mahieux, Catherine Daniel, Marie Titécat, Nicolas Beauval, Isabelle Houcke, Christel Neut, Delphine Allorge, Frédéric Borges, Gwénaël Jan, Benoît Foligné, Anne Garat

**Affiliations:** 1 U1286–INFINITE-Institute for Translational Research in Inflammation, Institut Pasteur de Lille, CHU Lille, Université de Lille, F-59000 Lille, France; fanny.george@univ-lille.fr (F.G.); severine.mahieux@univ-lille.fr (S.M.); marie.titecat@univ-lille.fr (M.T.); isabelle.houcke@univ-lille.fr (I.H.); christel.neut@univ-lille.fr (C.N.); 2 U1019-UMR 9017–Center for Infection and Immunity of Lille, Institut Pasteur de Lille, CHU Lille, Université de Lille, F-59000 Lille, France; catherine.daniel@ibl.cnrs.fr; 3 ULR 4483-IMPECS-IMPact de l’Environnement Chimique sur la Santé humaine, Institut Pasteur de Lille, CHU Lille, Université de Lille, F-59000 Lille, France; nicolas.beauval@chru-lille.fr (N.B.); delphine.allorge@univ-lille.fr (D.A.); anne.garat@univ-lille.fr (A.G.); 4 Unité fonctionnelle de Toxicologie, Institut Pasteur de Lille, CHU Lille, Université de Lille, F-59000 Lille, France; 5 LIBio, Université de Lorraine, F-54000 Nancy, France; frederic.borges@univ-lorraine.fr; 6 STLO, INRAE, Agrocampus Ouest, Institut Agro, Science & Technologie du Lait & de l’Œuf, F-35042 Rennes, France; gwenael.jan@inrae.fr

**Keywords:** bioremediation, gut microbiota, lactic acid bacteria, Enterobacterales, lead, cadmium, aluminum, probiotics, ICP-MS

## Abstract

Toxic metals (such as lead, cadmium, and, to a lesser extent, aluminum) are detrimental to health when ingested in food or water or when inhaled. By interacting with heavy metals, gut and food-derived microbes can actively and/or passively modulate (by adsorption and/or sequestration) the bioavailability of these toxins inside the gut. This “intestinal bioremediation” involves the selection of safe microbes specifically able to immobilize metals. We used inductively coupled plasma mass spectrometry to investigate the in vitro ability of 225 bacteria to remove the potentially harmful trace elements lead, cadmium, and aluminum. Interspecies and intraspecies comparisons were performed among the Firmicutes (mostly lactic acid bacteria, including *Lactobacillus* spp., with some *Lactococcus*, *Pediococcus*, and *Carnobacterium* representatives), Actinobacteria, and Proteobacteria. The removal of a mixture of lead and cadmium was also investigated. Although the objective of the study was not to elucidate the mechanisms of heavy metal removal for each strain and each metal, we nevertheless identified promising candidate bacteria as probiotics for the intestinal bioremediation of Pb(II) and Cd(II).

## 1. Introduction

Hazardous toxic metals (such as lead (Pb), cadmium (Cd), and, to a lesser extent, aluminum (Al)) are nonessential and nonbiodegradable elements that are detrimental for health through (i) poisoning after acute environmental exposure, (ii) long-term, low-dose contamination via the food chain, or (iii) inhalation. When considering the exposome in humans and other animals, heavy metals and other toxins can enter the body by the oral route after the inhalation or ingestion of contaminated drinking water, beverages, or food. After first interacting with the gastrointestinal tract and its ecosystem, the metals may accumulate further within target tissues and also reach the circulation [1,2].

Lead (Pb) is a widespread heavy metal considered to be “probably carcinogenic” by the US Environmental Protection Agency (EPA) and classified as a group 2A substance by the International Agency for Research on Cancer (IARC). Chronic Pb exposure leads to anemia, increased blood pressure, persistent vomiting, and neuropsychiatric disorders (including encephalopathy, delirium, convulsions, and even coma in severe cases) [3,4]. It is noteworthy that children are highly susceptible to Pb exposure, which may cause mental retardation [5]. Recent studies have suggested that Pb exposure in childhood is a risk factor for the development of neurodegenerative diseases in adulthood [6]. Pb accumulates mostly in the liver, kidneys, and bones. Safe drinking water should contain less than 10 μg.L^−1^ Pb—a threshold that is very often exceeded [7].

Cadmium (Cd) is classified as a group I carcinogenic compound by the IARC [8]. The metal is nephrotoxic and may induce various health disorders, such as damage to bones (osteoporosis), kidney tubules, the brain, and the testis [9,10,11]. It has also been suggested that Cd is involved in metabolic diseases and susceptibility to respiratory tract infections [12,13]. A Cd intake of 23.2 μg/day (less than half the safe intake, according to current guidelines) might increase the risk of chronic kidney disease, mortality from heart disease, cancer at any site, and Alzheimer’s disease [14]. Moreover, epidemiological studies of co-exposure to Cd and Pb have shown that each metal enhances the nephrotoxicity of the other. Furthermore, Cd is also involved in the modulation of inflammatory responses [15], including those in the gastrointestinal tract [16]. Perinatal and early-life exposure to Cd has been linked to poor birth outcomes and adverse effects on the child’s neurodevelopmental and metabolic functions [17].

Aluminum (Al) has no known physiological functions and accumulates in the liver, kidneys, bones, testis, and (where it produces the most prominent toxic effects) the brain and the nervous system [18,19]. It has also been suggested that Al is involved in neurological disorders, such as Alzheimer’s disease, autism spectrum disorders, and multiple sclerosis [20]. With regard to the digestive tract, Al induces epithelial barrier dysfunction, abdominal pain, and inflammation [21,22], and the metal’s involvement in inflammatory bowel disease is strongly suspected [23,24,25]. Al adversely affects reproduction [25], and in utero exposure has a negative impact even at low concentrations [26].

Furthermore, ingested metal xenobiotics may also contribute to dysbiosis by targeting the host’s gut microbiota and the latter’s key functional roles in intestinal homeostasis [16,27]. Hence, metal contaminants may indirectly alter the host’s health as a result of subtle microbial changes within the gut [28,29]; these changes might damage the intestine’s barrier functions and might contribute to a broad range of metabolic, neurological, and/or chronic immune diseases. Indeed, microbes interact (by biotransformation or sequestration) with metals in the gut, and thus can actively or passively, control bioaccessibility and the further bioavailability of heavy metals [30]. In experiments on germ-free mice [2,31] or involving broad-spectrum antibiotics [32], we and others have previously highlighted the gut microbiota’s overarching role as a barrier to heavy metal dissemination. However, not all bacteria have the same ability to limit the bioavailability of toxic metals. Whereas the use of environmental bacteria as biosorbents for heavy metals has been widely employed to remove metals from contaminated soils and wastewaters [33], “intestinal bioremediation” requires the selection of safe microbes on the basis of their specific ability to immobilize metals [34]. Given their ecological niches, food-grade bacteria and gut-sourced microorganisms are thus the best candidates for alleviating metal toxicity [35]. In this context, lactic acid bacteria (LAB) efficiently bind and/or internalize metals (especially Cd and Pb) in vitro [36,37,38,39,40].

Although LAB-mediated metal removal is partly strain-dependent, few researchers have explored species and strain diversity in this respect. Overall, only a few types of LAB (lactobacilli, enterococci, and *Weissella* spp. [40,41,42]), dairy propionibacteria, and bifidobacteria [38,43] have been analyzed in separate, heterogenous studies. Furthermore, non-LAB have rarely been screened for these properties: only a small number of proteobacterial species (*E. coli*) and gut-isolated anaerobic bacteria (*Akkermansia muciniphila*, *Faecalibacterium prausnitzii*, and *Oscillibacter ruminantium* single strain isolates) have been studied [32,43]. Other genera with notable in vitro detoxification potential (such as *Pseudomonas*, *Stenotrophomonas*, or *Bacillus*) are not appropriate for targeting the intestinal compartment. To date, only LAB such as *Lactobacillus plantarum*, *Lactobacillus casei*, *Lactobacillus rhamnosus*, and *Lactobacillus delbrueckii* strains have been selected in vitro and then confirmed as being able to detoxify in vivo. Various selected food-sourced microbes can prevent the absorption of heavy metals in the gut (and thus dissemination into the tissues) and thus enable their excretion in the feces. The efficacy of this approach has been demonstrated in preclinical studies of acute and chronic Pb [32,44,45], Cd [46,47], and Al [48,49] toxicity in the mouse.

Among probiotic lactobacilli, cell-surface-associated compounds are responsible for the strains’ functional specificity [50]. The mechanism by which metals bind to the bacterial cell wall is thought to depend on the huge variety of surface molecules on individual bacterial species and strains [51], including teichoic and lipoteichoic acids and peptidoglycans. One can therefore hypothesize that the distinct biosorbent properties of other Gram-positive and Gram-negative bacteria depend on binding sites such as S-layer proteins, cell surface proteins, and polysaccharides.

Here, we assessed interspecies and interstrain variability in the ability to remove potentially harmful Pb, Cd, and Al in vitro. The study’s primary objective was to compare intrinsic aptitudes to cope with heavy metals among bacteria from the Firmicutes, Actinobacteria, and Proteobacteria, rather than to elucidate the mechanisms of metal biosorption or bioaccumulation per se (e.g., plotting adsorption isotherms). We studied many LAB (*n* = 99), several bifidobacteria (*n* = 11), dairy propionibacteria (*n* = 21), and cutibacteria (*n* = 4), together with other gut-friendly bacteria such as the Enterobacterales (*n* = 90). The study’s secondary objective was to identify the best candidates for use in preclinical assays and further veterinary and clinical applications.

## 2. Materials and Methods

### 2.1. Chemicals, Reagents and Instruments

Chemicals and reagents were purchased from Sigma-Aldrich Chemical (Saint-Quentin-Fallavier, France), unless otherwise stated. Ultrapure water corresponds to PURELAB Option-Q from Veolia Water (Antony, France). The Ultraflex III matrix-assisted laser desorption/ionization time-of-flight/time-of-flight (MALDI-TOF/TOF) instrument and Flex Analysis software were from Bruker Daltonik GmbH (Bremen, Germany). Metal concentrations in diluted samples were determined using inductively coupled plasma mass spectrometry (ICP-MS; THERMO ICAP Qc, Thermo Scientific, Courtaboeuf Cedex, France).

### 2.2. Bacterial Strains Collections and Culture Conditions

We studied a set of 225 bacterial strains from various sources. Most of the LAB came from the well-characterized DSM and ATCC collections previously used for comparative genomics studies of lactobacilli and associated genera [52].

The Propionibacteria sample consisted of 21 *Propionibacterium freudenreichii* strains from the Centre International de Ressources Microbiennes-Bactéries d’Intérêt Alimentaire collection (CIRM-BIA; STLO, Institut National de Recherche pour l’Agriculture, l’Alimentation et l’Environnement, Rennes, France), previously characterized for their immunomodulatory potential using comparative genomics [53].

Most of the *Escherichia coli* strains belonged to the *Escherichia coli* ECOR standard reference collection [54]. The later includes isolates (A, B1, B2, D, and E phylogroups) from a variety of hosts and geographic regions and were kindly provided by Dr. Laurent Debarbieux (Institut Pasteur, Paris, France). Other *E. coli*-type strains and adherent invasive *E. coli* pathovars (AIEC) have been described previously [55]. Strains of *Serratia marcescens* (Db10, JUb9, SM25, SM38, and SM45) were kindly provided by Dr. Elizabeth Pradel (Institut Pasteur de Lille, Lille, France) [56]. Some cheese-derived *Hafnia alvei* strains (Gb01, E215, 920 and Grignon) have been described elsewhere [57]. Lastly, a few bacterial strains (nine *Bifidobacterium* species, four *Cutibacterium acnes*, two *Enterobacter,* two *Hafnia alvei*, and five *Klebsiella*) were sourced from historical clinical gut or fecal samples of human origin, from food, or as re-isolates from commercial probiotic products (Bb12 and Morinaga) held in a collection at the Faculty of Pharmacy of Lille (FPL collection), University of Lille, France. The strains were identified using selective media and then MALDI-TOF and were denoted by internal FPL numbers.

Strains of *Lactobacillus* and associated genera (*Fructobacillus*, *Leuconostoc*, *Lactococus*, *Pediococcus*, and *Weissella*) were cultured without shaking in de Man, Rogosa, and Sharpe (MRS) medium, and *Carnobacterium* and *Staphylococcus* were cultured in BHI (brain heart infusion) at 30 or 37 °C, depending on their optimal growth temperature. Bifidobacteria were grown anaerobically using anaerobic generator packs (GENbaganaer, Biomérieux, France) in MRS supplemented with 0.1% (*w/v*) L-cysteine hydrochloride. *P. freudenreichii* strains were grown at 30 °C under microaerophilic conditions and without shaking in yeast extract lactate medium [58]. Strains of Enterobacterales (*Enterobacter*, *Escherichia*, *Hafnia, Klebsiella*, and *Serratia*) were grown in Luria–Bertani medium at 37 °C without shaking. Depending on the bacterial strain, the stationary phase was achieved after 12 to 72 h of culture.

### 2.3. Metal Removal Assays

Eight mL of a stationary-phase bacterial culture was standardized at an optical density at 600 nm of 2.5 and then washed twice in Ringer’s solution. The pellet was suspended with 8 mL of the corresponding metal ion solution (in Ringer’s, pH 7.0) containing 25 ppm (PbCl_2_ or AlCl_2_) or 1 ppm CdCl_2_ and then gently mixed using a rotary agitator (12 rpm) at room temperature for 1 h. Samples were then centrifuged and washed twice before quantification of the amount of metal using ICP-MS. The pellets were suspended in 500 μL of 70% nitric acid and heated at 98 °C for 15 min. Lastly, the samples were diluted in mQ water and assayed using ICP-MS. For each strain, the residual metal mass in the pellet was expressed as a percentage of the initial amount in the incubation medium. All assays were performed in triplicate, corresponding to three distinct bacterial cultures. The bacteria–metal incubation time was set to one hour in order to mimic the food transit time and thus the possible contact time in the gut.

We first checked that our methods were reliable and appropriate for screening distinct bacterial strains for their ability to remove Pb, Cd, or Al. It is known that many factors influence the levels of metal binding by bacteria: the contact time, temperature, pH, metal ion concentration, washing buffer, and inoculum size [36,59,60]. Thus, several key parameters were defined to either mimic the gut environment or for reasons of convenience, e.g., the incubation temperature. The binding assay was thus performed in physiological saline solution (Ringer’s solution) at a neutral pH (7.1) and at room temperature (22 ± 2 °C), using time-separated (triplicate) cultures to test the reproducibility. Pb and Al concentration at 25 ppm were selected as being realistic for evaluating metal sequestration, whereas a lower concentration of Cd (1 ppm) was required for discrimination between strains. In fact, 25 ppm Cd did not discriminate between strains—most of which had very low Cd binding capacity (removal < 5%) in an initial prescreen. Consequently, only the most promising strains (removal > 5%) were assessed at 1 ppm. It is noteworthy that the selected concentrations of Pb, Cd, and Al were similar to those reported previously [40,43,49,51]. We therefore ranked a strain’s removal capacity as “weak”, “low”, “moderate”, or “high” (respectively, 0–25%, 26–50%, 51–75%, and 76–100% for Pb, and 0–10%, 11–20%, 21–30%, and >30% for Cd and Al).

The screening assay was based on the percentage of metal bound strongly to the pelleted bacteria. However, we also confirmed the stability and irreversibility of binding in order to further discriminate between strains that bound both Pb and Cd. To this end, we performed two serial wash cycles (resuspension of the pellet in a metal-free solution and then centrifugation). In fact, the residual quantities of metal in the second and last wash samples were barely detectable or undetectable. This is illustrated by selected examples of strains with different binding efficiencies (Figure S1A,B). However, the Al binding was more labile and appeared to be partly reversible after rinsing, although the serial wash cycle was quite reproducible (Figure S1C). Consequently, our assay somewhat overestimated Al binding but enabled us to discriminate between strains; the assay result might reflect the bacteria’s intrinsic ability to interact with the metal under physiological conditions, i.e., in the gastrointestinal tract.

### 2.4. Statistical Analyses

All graphical and statistical analyses were performed with GraphPad Prism software (version 6.0, GraphPad Software Inc., San Diego, CA, USA). Experimental groups were compared with their respective controls in a nonparametric, one-way analysis of variance (the Mann–Whitney U test) or a two-tailed Student’s *t*-test, as appropriate. Quantitative variables were quoted as the mean ± standard deviation (SD). Data with *p* values ≤ 0.05 were considered to be significant.

## 3. Results

### 3.1. Lactic Acid Bacteria (LAB) Exhibit Variable Pb Removal Capacities

**Figure 1:**
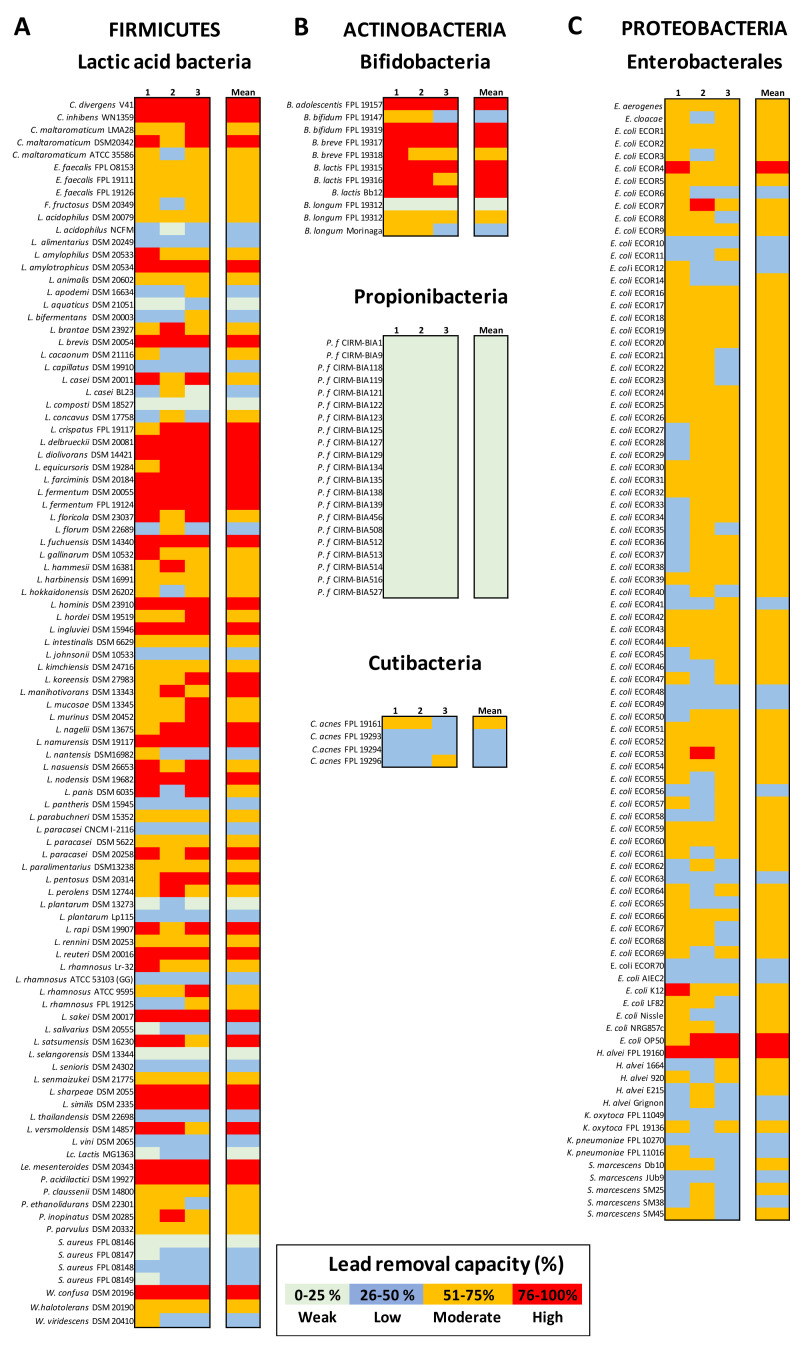
The Pb(II) removal capacity of bacteria from distinct taxonomic phyla. Panel (**A**): Lactic acid bacteria (LAB). Panel (**B**): Actinobacteria, comprising bifidobacteria, propionibacteria, and cutibacteria. Panel (**C**): Proteobacteria, such as the Enterobacterales. Metal removal capacity is defined as the mean ± SD percentage of the initial quantity of metal in solution, in triplicate experiments. Here, the Pb removal capacity is color-coded as weak (0 to 25%: pale green), low (26 to 50: blue), moderate (51 to 75%: orange), or high (76 to 100%: red).

**Figure 2:**
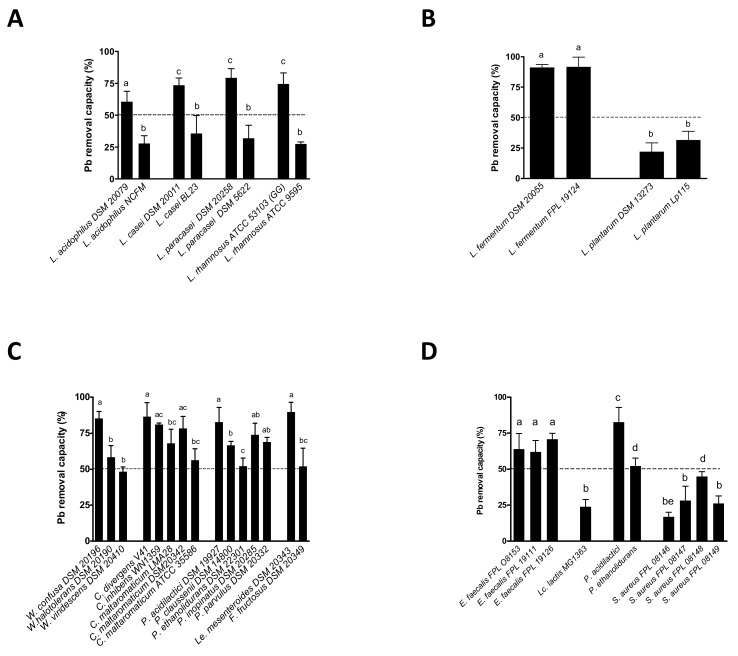
The Pb(II) removal capacity of distinct strains of LAB. Panel (**A**): Five selected pairs of strains from the same species but with distinct removal capacities. Panel (**B**): Two selected pairs of strains from the same species but with similar removal capacities. Panel (**C**): Four selected groups of strains from the same genus but with distinct removal capacities. Panel (**D**): Four selected strains from groups of cocci with distinct removal capacities. Metal removal capacity was defined as the mean ± SD percentage of the initial quantity of metal in solution, in triplicate experiments. Different letters indicate significant (*p* < 0.05) between-strain differences.

Among the 99 individual LAB strains tested for their capacity to remove Pb(II) salts at 25 ppm, over two-thirds were able to immobilize between 50% and 90% of the metal in solution (Figure 1A). When considering only the genus *Lactobacillus* (covering 65 distinct species and 76 strains), the removal capacity of Pb ranged from 6% ± 2.5 to 92% ± 8.5. The species-level patterns were inconsistent: marked interstrain differences could be observed for given species, such as *L. acidophilus*, *L. casei*, *L. paracasei* and *L. rhamnosus* (Figure 2A). The two *L. fermentum* strains were particularly effective for Pb removal, whereas the two *L. plantarum* strains were unexpectedly poor (Figure 2B).

The Pb removal capacity also varied within other species of LAB. *Carnobacterium* spp., *Pediococcus* spp., *Leuconostoc*, *Fructobacillus*, and *Weissella* spp. demonstrated high Pb removal capacities (Figure 2C). This potential was not related to the bacteria’s shape (i.e., bacilli vs. cocci); the three enterococci and four pediococci tested were quite good Pb biosorbers (>50%), whereas *Lactococcus lactis* and four distinct strains of *Staphylococcus aureus* were not (mostly <30%) (Figure 2D).

### 3.2. Actinobacteria Strains Differ in Their Pb Biosorption Potential

Within the Actinobacteria, the bifidobacteria’s Pb removal capacities ranged from weak (e.g., 6.6%) to high (e.g., 90%), depending on the strain (Figure 1B). Interstrain differences were observed for all species; for example, some *Bifidobacterium longum* strains had a removal capacity of up to 10-fold more than others (i.e., 6.75% ± 0.9 vs. 65.4% ± 7.2; *p* < 0.001). Surprisingly, none of the *Propionibacterium freudenreichii* strains of dairy origin were able to remove much Pb (mean: 10.25% ± 4.4). In contrast, four *Cutibacterium acnes* strains (previously referred to as *Propionibacterium acnes*) had removal capacities of 41%, 44%, 49%, and 56.8%, respectively.

### 3.3. Enterobacterales Have a Moderate Pb Biosorption Potential

We evaluated 90 strains belonging to the class of Gammaproteobacteria and to the order of Enterobacterales, comprising 68 *E. coli* strains from the ECOR library, extended to five other *E. coli* strains showing either probiotic properties (*E. coli* Nissle 1917), pathobiont traits such adherent and invasive capacities, e.g., LF82 and NRG857C, or no particular criterion from a physiological point of view (*E. coli* K12). Furthermore, other genera were considered, with five dairy isolates of *Hafnia alvei*, two *Klebsiella* spp., two *Enterobacter* spp., and five *Serratia marcescens* strains from clinical collections, as commensal prototypes. Overall, the Enterobacterales strains’ ability to remove Pb was moderate and uniform (54.14 ± 6.7%). Indeed, nearly 90% of the strains of Gram-negative bacilli had Pb removal capacities between 45% and 65%. Only two *E. coli* strains and a single *Hafnia alvei* strain were able to immobilize more than 75% of the Pb in a 25 ppm solution.

### 3.4. Bacterium-Mediated Cd Removal Capacity Is Phylum-, Genus-, and Strain-Specific

**Figure 3:**
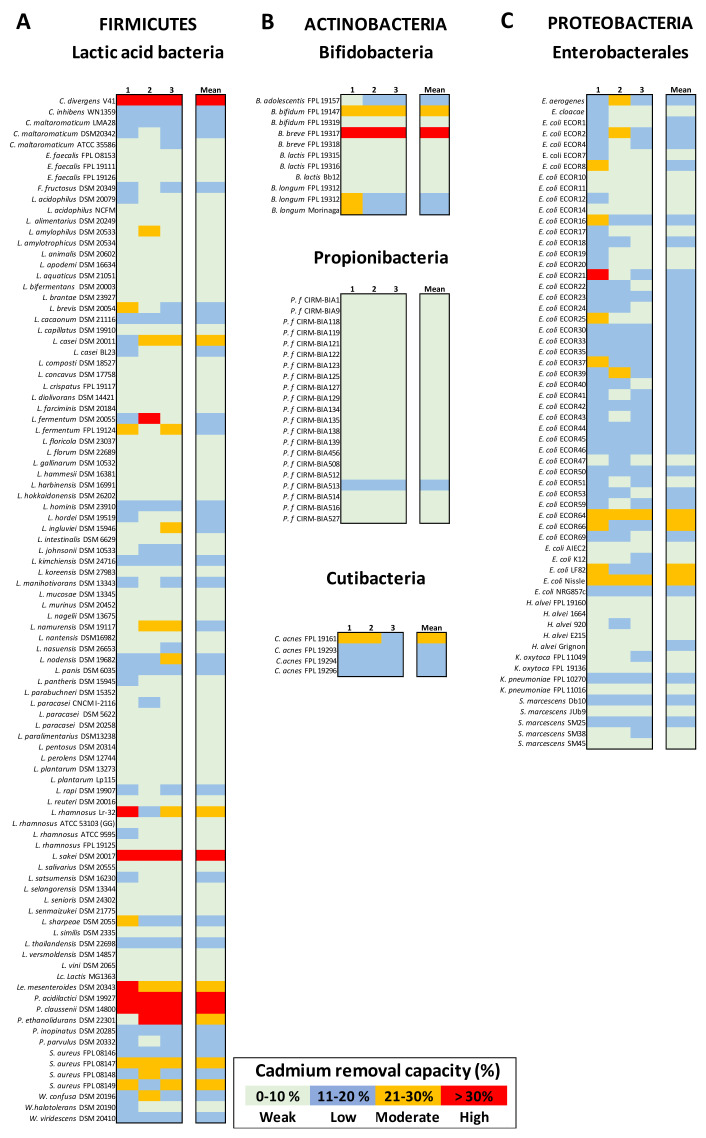
The Cd(II) removal capacity of bacteria from distinct taxonomic phyla. Panel (**A**): LAB. Panel (**B**): Actinobacteria, comprising bifidobacteria, propionibacteria, and cutibacteria. Panel (**C**): Proteobacteria, as Enterobacterales. Metal removal capacity was defined as the mean ± SD percentage of the initial quantity of metal in solution, in triplicate experiments. Here, the Cd removal capacity is color-coded as weak (0 to 10%: pale green), low (11 to 20%: blue), moderate (21 to 30%: orange), or high (over 30%: red).

As explained in the Methods section, the Cd concentration of 25 ppm was not appropriate for discriminating between bacterial strains with respect to Cd(II) binding or for mimicking real intoxication events. Hence, we tested the bacteria’s ability to remove Cd from a 1 ppm solution. Among the 95 LAB strains tested, (90%) removed Cd weakly (<20% binding; Figure 3A). Interestingly, a few strains (three *Pediococcus* spp., a *Carnobacterium divergens* strain, and, to a lesser extent, a *L. rhamnosus* strain and a *Leuconostoc mesenteroides* strain) had Cd binding capacities over 25% and even up to 50% ± 15.7 for a *Pediococcus acidilactici* isolate.

When considering the Actinobacteria, the bifidobacteria were characterized by variable Cd removal that depended more on the strain than on the species. Indeed, the removal capacity of *Bifidobacterium breve* strains ranged from 6.2% ± 0.6 to 40.7% ± 6.7 (*p* < 0.01), and that of *B. longum* strains ranged from 3.6% ± 1.7 to 18.9% ± 5.8 (*p* < 0.01). Most of the *Propionibacterium freudenreichii* strains were consistently weak Cd biosorbers, whereas *C. acnes* strains removed Cd poorly or moderately (Figure 3B). Lastly, near all the 60 Enterobacterales tested were weak or low Cd chelators, with the exception of four *E. coli* strains with a moderate Cd removal potential (ECOR 64, ECOR 66, *E. coli* LF82, and *E. coli* Nissle, with values of 24.2% ± 3.6, 21.2% ± 5.6, 20.5% ± 2.2, and 25.2% ± 3.5, respectively) (Figure 3C).

### 3.5. Bacteria-Mediated Al Removal Capacity Is Also Genus- and Strain-Dependent

**Figure 4:**
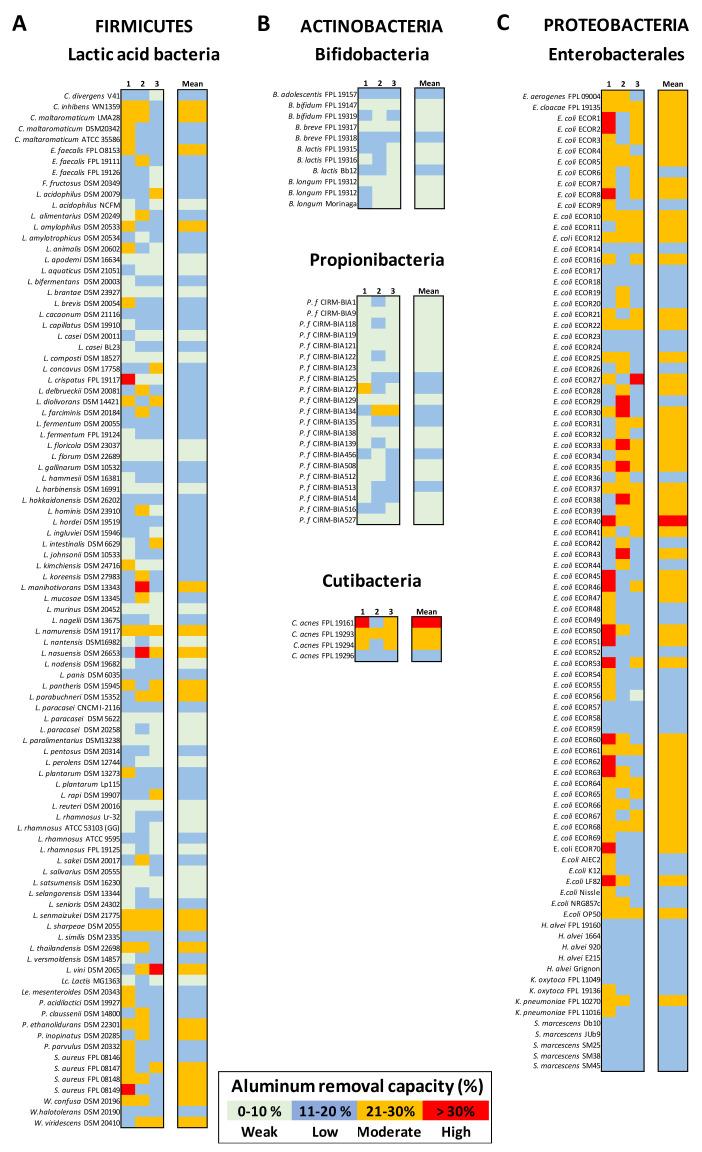
Al(III) removal capacity of bacteria from distinct taxonomic phyla. Panel (**A**): LAB. Panel (**B**) Actinobacteria, comprising bifidobacteria, propionibacteria, and cutibacteria. Panel (**C**): Proteobacteria, such as the Enterobacterales. Metal removal capacity was defined as the mean ± SD percentage of the initial quantity of metal in solution, in triplicate experiments. Here, the Al removal capacity is color-coded as weak (0 to 10%: pale green), low (11 to 20%: blue), moderate (21 to 30%: orange), or high (over 30%: red).

The bacteria’s ability to remove Al(III) in AlCl_3_ solution depended on the strain’s origin and phylogenic diversity. The LAB’s ability to remove Al from a 25 ppm solution differed from one species to another; it ranged from 5% to 28%, with an average of 14.8% ± 4.7 (Figure 4A). Similarly, bifidobacteria and propionibacteria bound Al quite weakly (mean: 8.9% ± 2.8 and 9.4% ± 2.6, respectively), and the values rarely exceeded 10%. Strains of *Cutibacterium acnes* were more effective, with moderate removal capacities (mean: 24.3% ± 4.7) (Figure 4B). In contrast, representatives of Enterobacterales had removal capacities that ranged from 12% to 30%, with a mean value of 20.4 ± 4.7% (Figure 4C). A few *E. coli* strains removed Al strongly (i.e., 25% to 30% for ECOR37, ECOR40, ECOR50, and ECOR64). The *Hafnia* and *Serratia* strains were less effective, with values below 15%.

### 3.6. The Ability of Bacteria to Remove Pb and Cd Is Not Greatly Affected When Both Metals Are Present

**Figure 5:**
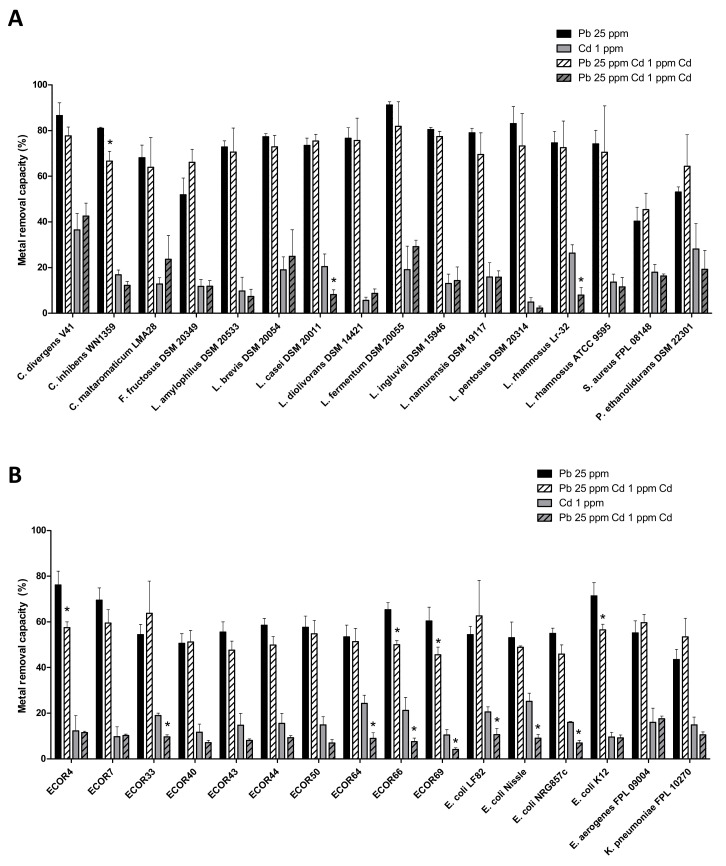
Pb removal capacities for 25 ppm Pb alone (black) or with 1 ppm Cd (hatched black) and Cd removal capacities for 1 ppm Cd alone (in grey) or with 25 ppm Pb (hatched grey). Panel (**A**): Lactic acid bacteria and other Gram positive bacteria. Panel (**B**): Enterobacterales. Metal removal capacity was defined as the mean ± SD percentage of the initial quantity of metal in solution, in triplicate experiments. * indicates a statistically significant (*p* < 0.05) difference between the metal alone and the metal in a mixture.

Given that co-exposure to Pb and Cd is commonly (due to their co-occurrence in food, water, and the environment more generally), we also assayed removal capacities when the two metals were present (i.e., a solution containing 25 ppm Pb and 1 ppm Cd) for 16 arbitrarily selected Gram-positive bacteria (Figure 5A) and 16 arbitrarily selected Gram-negative bacteria (Figure 5B). Interestingly, the bacteria’s removal capacity for one metal was not greatly influenced by the presence of the other—except for few strains showing a relative decrease of 20% to 40% vs. the metal alone.

## 4. Discussion

Here, we addressed the Pb, Cd, and Al removal capacity of more than 200 bacterial strains; these were mostly LAB, associated genera, and representative gut enterobacteria. It is noteworthy that all the tested bacteria were able to survive at the studied metal concentrations (i.e., 25 ppm for Pb and Al and 1 ppm for Cd), as previously described elsewhere for several LAB strains [40]. Indeed, all the tested strains survived exposure to Pb concentrations of more than 1000 ppm. The strains’ respective Cd removal capacities were not correlated with the (higher) minimal inhibitory concentration (MIC). When considering strains with a MIC of 34.5 ppm (for example), the Cd removal capacity ranged from 1.5% to 23.6% (Figure S2). This observation suggests that metal tolerance is not a guide to metal removal, as has also been demonstrated for Pb with various *L. plantarum* strains [61]. Live bacteria are not necessarily required for significant metal biosorption. Indeed, binding isotherms in the Langmuir model showed that the maximum binding capacity (*Q_max_*) was high for both boiled (dead) and live forms of two probiotic strains (*Lactobacillus rhamnosus* and *Propionibacterium freudenreichii*) [36]. Other researchers have demonstrated that dead and live bacteria have similar binding capacities for Pb and for Cd [32,36,41,42]. However, live forms are slightly more effective for Pb removal as a result of cell-specific intracellular metal accumulation [62,63].

Approaches based on the lactobacilli’s surface characteristics (such as hydrophobicity and electrostatic properties) failed to identify relevant selection criteria for Pb and Cd removal [64]. Indeed, we currently lack hypothesis-driven criteria for selecting strains with a high detoxification capacity. In the present hypothesis-free study, we characterized the removal metal capacity of live bacteria with regard to species and strain diversity. We used gut-friendly (nonpathogenic) bacteria sourced from food for from intestinal ecological niches. Most of these bacteria are “generally regarded as safe” by the US Food and Drug Administration—the GRAS status and safety in general being an essential characteristic for further in vivo applications. In contrast to many studies of metal removal from solutions in deionized water, we used neutral, isotonic Ringer’s solution for the binding assays and the washing cycles.

Our present results for metal removal confirm and extend the broad spectrum of functional diversity observed among Gram-positive and Gram-negative bacteria. The mechanisms of metal removal are strain-dependent and have been described elsewhere [52,65]; they include ion exchange, chelation, adsorption by physical forces, and intracellular sequestration. The role of hydroxyl (from the peptidoglycan), carboxyl, and phosphate (surface protein) groups is influenced by pH, specificity, and abundance; along with capsular polysaccharides, these are assumed to be the key determinants of metal binding. Thus, a cell’s overall removal capacity is a complex, multifactorial variable. Although many variables (e.g., culture conditions, media, and growth phase) are independent of the individual bacterial genes, comparative genomic studies of LAB might help to identify specific genes that are up- or downregulating factors involved in metal removal.

We found that LAB and bifidobacteria have generally moderate to high Pb removal capacities, whereas dairy propionibacteria consistently have weak capacities. Gram-negative bacteria have almost low to moderate Pb removal capacities. We were able to identify some good candidates for Pb removal among the lactobacilli and bifidobacteria, as previously described for *Lactobacillus sakei*, *L actobacillus delbruckii*, *L actobacillus fermentum* and *Bifodobacterium bifidum* strains [44]. In line with the literature data, we found that *Weissella* and *Pediococcus* spp. had high Pb removal capacities [40]. To the best of our knowledge, the present study is the first to have described the high Pb removal capacity of *Carnobacterium* spp.

With regard to Cd, five of the 220 strains (one *B. breve* strain, one *L. sakei* strain, one *Carnobacterium divergens* strain and two *Pediococcus* strains) had a high removal capacity—suggesting that some LAB not generally considered to be probiotics may have valuable properties for Cd bioremediation purposes. In contrast to previous reports [44,47], the few *L. rhamnosus* and *L. plantarum* strains in our study exhibited poor Pb and Cd removal capacities. Again, it should be borne in mind that metal removal capacities are highly strain-dependent and species-level generalizations cannot be made. Interestingly, all 20 strains of *Propionibacterium* had similar but low removal capacities for both Pb and Cd, despite the fact that they have very strain-specific surface protein and exopolysaccharide profiles (related to various immunological properties) [65].

There are few published data on Al removal by bacteria. A literature study of *L. plantarum* and *L actobacillus reuteri* strains [49] with a similar experimental design found a higher removal capacity (25%) than we did. It is noteworthy that the Al removal capacity was consistently higher in *Enterobacteria* than in LAB and *Actinobacteria*.

Interestingly, we found that the bacteria’s ability to remove Pb and Cd is not greatly affected when the metals are mixed. This encourages us to select strains able to remove both Pb and Cd to a large extent. It remains to be seen whether or not mixing strains has synergistic effects on Pb and Cd removal [66].

It is proven that bacterial strains with a high metal removal capacity in vitro can also have this potential in in vivo models of acute and chronic poisoning in mice [32,44,45,46,47,49], rats [66], and humans [67]. The initial screening described here must now be extended in vivo in order to take account of the physiology of the gastrointestinal tract and the presence of other essential metals, trace elements, and organic molecules. Heavy metal influences the gut microbiota’s structure, diversity, and function [28] (including heavy metal sequestration), and prebiotics can interfere with the equilibria of heavy metals too [68]. Thus, the bidirectional relationship of dysbiosis and heavy metals in various pathologies and the use of probiotics are highly complex [27] and will have to be taken into account when developing personalized medicine treatments [69]. Lastly, the strength of metal binding under physiological conditions remains to be determined. Although the bacteria can absorb heavy metals in preclinical models of acute and chronic exposures, possible interactions with food matrices must be addressed. Interestingly, bacteria with good heavy metal removal capacities can also be incorporated into fermented foods. In vitro simulations of gastrointestinal digestion might provide this information and might help to mimic the bioremediation process in the presence of various foods. Nonetheless, it might be possible to use selected exogenous food-grade bacteria to lower heavy metal levels, just as chemical chelators or food-derived fibers can be used to treat metal poisoning. This approach might enable the development of specific probiotics or probiotic-fermented foods for countering exposure to xenobiotics.

## 5. Conclusions

Taken as a whole, our present results reveal that bacteria are highly diverse in their ability to remove Pb, Cd, Al, or a mixture of Pb and Cd in vitro. By exploring the interspecies and interstrain diversity of LAB, bifidobacteria, propionibacteria, and enterobacteria, we found that bacterial metal removal is strain- and metal-dependent. These results open up several perspectives for further research. Firstly, it may be possible to identify probiotic candidates with a long history of safe use for the human or veterinary treatment of acute heavy metal poisoning or chronic heavy metal contamination, in combination with conventional chelation, antioxidant, and anti-inflammatory therapies. Secondly, our results may help to understand the role of gut-resident microbes in modulating toxic metal levels.

## Data Availability

The data presented in this study are available on request from the corresponding author.

## Supplementary Materials

The following are available online at https://www.mdpi.com/2076-2607/9/2/456/s1. Figure S1: Selected examples of bacterial strains with distinct metal removal capabilities, demonstrating the accuracy of the assays for Pb (A), Cd (B), and Al (C). Metals were quantified in the binding supernatant, in the two washing buffers, and in the final bacterial pellet. Figure S2: Selected examples of bacterial strains with equivalent MICs for Cd but different Cd removal capacities, illustrating the lack of a relationship between metal resistance and metal binding. MICs were determined in 24-hr liquid cultures. The Cd removal capacity is color-coded as weak (0 to 10%: pale green), low (11 to 20%: blue), and moderate (21 to 30%: orange).

## References

[1] Breton J., Le Clère K., Daniel C., Sauty M., Nakab L., Chassat T., Dewulf J., Penet S., Carnoy C., Thomas P. (2013). Chronic Ingestion of Cadmium and Lead Alters the Bioavailability of Essential and Heavy Metals, Gene Expression Pathways and Genotoxicity in Mouse Intestine. Arch. Toxicol..

[2] Breton J., Daniel C., Dewulf J., Pothion S., Froux N., Sauty M., Thomas P., Pot B., Foligné B. (2013). Gut Microbiota Limits Heavy Metals Burden Caused by Chronic Oral Exposure. Toxicol. Lett..

[3] Flora G., Gupta D., Tiwari A. (2012). Toxicity of Lead: A Review with Recent Updates. Interdiscip. Toxicol..

[4] Wani A.L., Ara A., Usmani J.A. (2015). Lead Toxicity: A Review. Interdiscip. Toxicol..

[5] Lanphear B.P., Hornung R., Khoury J., Yolton K., Baghurst P., Bellinger D.C., Canfield R.L., Dietrich K.N., Bornschein R., Greene T. (2005). Low-Level Environmental Lead Exposure and Children’s Intellectual Function: An International Pooled Analysis. Environ. Health Perspect..

[6] Reuben A. (2018). Childhood Lead Exposure and Adult Neurodegenerative Disease. J. Alzheimers Dis. JAD.

[7] Watt G.C., Britton A., Gilmour H.G., Moore M.R., Murray G.D., Robertson S.J. (2000). Public Health Implications of New Guidelines for Lead in Drinking Water: A Case Study in an Area with Historically High Water Lead Levels. Food Chem. Toxicol. Int. J. Publ. Br. Ind. Biol. Res. Assoc..

[8] Iternational Agency for Research on Cancer (1993). Cadmium and Cadmium Compounds. IARC Monogr. Eval. Carcinog. Risks Hum..

[9] Järup L., Akesson A. (2009). Current Status of Cadmium as an Environmental Health Problem. Toxicol. Appl. Pharmacol..

[10] Rinaldi M., Micali A., Marini H., Adamo E.B., Puzzolo D., Pisani A., Trichilo V., Altavilla D., Squadrito F., Minutoli L. (2017). Cadmium, Organ Toxicity and Therapeutic Approaches: A Review on Brain, Kidney and Testis Damage. Curr. Med. Chem..

[11] Satarug S., Garrett S.H., Sens M.A., Sens D.A. (2010). Cadmium, Environmental Exposure, and Health Outcomes. Environ. Health Perspect..

[12] Buha A., Đukić-Ćosić D., Ćurčić M., Bulat Z., Antonijević B., Moulis J.-M., Goumenou M., Wallace D. (2020). Emerging Links between Cadmium Exposure and Insulin Resistance: Human, Animal, and Cell Study Data. Toxics.

[13] Skalny A.V., Lima T.R.R., Ke T., Zhou J.-C., Bornhorst J., Alekseenko S.I., Aaseth J., Anesti O., Sarigiannis D.A., Tsatsakis A. (2020). Toxic Metal Exposure as a Possible Risk Factor for COVID-19 and Other Respiratory Infectious Diseases. Food Chem. Toxicol. Int. J. Publ. Br. Ind. Biol. Res. Assoc..

[14] Satarug S., Gobe G.C., Vesey D.A., Phelps K.R. (2020). Cadmium and Lead Exposure, Nephrotoxicity, and Mortality. Toxics.

[15] Hossein-Khannazer N., Azizi G., Eslami S., Alhassan Mohammed H., Fayyaz F., Hosseinzadeh R., Usman A.B., Kamali A.N., Mohammadi H., Jadidi-Niaragh F. (2020). The Effects of Cadmium Exposure in the Induction of Inflammation. Immunopharmacol. Immunotoxicol..

[16] Breton J., Daniel C., Vignal C., Body-Malapel M., Garat A., Plé C., Foligné B. (2016). Does Oral Exposure to Cadmium and Lead Mediate Susceptibility to Colitis? The Dark-and-Bright Sides of Heavy Metals in Gut Ecology. Sci. Rep..

[17] Sanders A.P., Claus Henn B., Wright R.O. (2015). Perinatal and Childhood Exposure to Cadmium, Manganese, and Metal Mixtures and Effects on Cognition and Behavior: A Review of Recent Literature. Curr. Environ. Health Rep..

[18] Becaria A., Campbell A., Bondy S.C. (2002). Aluminum as a Toxicant. Toxicol. Ind. Health.

[19] Igbokwe I.O., Igwenagu E., Igbokwe N.A. (2019). Aluminium Toxicosis: A Review of Toxic Actions and Effects. Interdiscip. Toxicol..

[20] Exley C., Clarkson E. (2020). Aluminium in Human Brain Tissue from Donors without Neurodegenerative Disease: A Comparison with Alzheimer’s Disease, Multiple Sclerosis and Autism. Sci. Rep..

[21] Jeong C.H., Kwon H.C., Kim D.H., Cheng W.N., Kang S., Shin D.-M., Yune J.H., Yoon J.E., Chang Y.H., Sohn H. (2020). Effects of Aluminum on the Integrity of the Intestinal Epithelium: An in Vitro and in Vivo Study. Environ. Health Perspect..

[22] Esquerre N., Basso L., Dubuquoy C., Djouina M., Chappard D., Blanpied C., Desreumaux P., Vergnolle N., Vignal C., Body-Malapel M. (2019). Aluminum Ingestion Promotes Colorectal Hypersensitivity in Rodents. Cell. Mol. Gastroenterol. Hepatol..

[23] Lerner A. (2007). Aluminum Is a Potential Environmental Factor for Crohn’s Disease Induction: Extended Hypothesis. Ann. N. Y. Acad. Sci..

[24] Pineton de Chambrun G., Body-Malapel M., Frey-Wagner I., Djouina M., Deknuydt F., Atrott K., Esquerre N., Altare F., Neut C., Arrieta M.C. (2014). Aluminum Enhances Inflammation and Decreases Mucosal Healing in Experimental Colitis in Mice. Mucosal Immunol..

[25] Vignal C., Desreumaux P., Body-Malapel M. (2016). Gut: An Underestimated Target Organ for Aluminum. Morphol. Bull. Assoc. Anat..

[26] Röllin H.B., Nogueira C., Olutola B., Channa K., Odland J.Ø. (2018). Prenatal Exposure to Aluminum and Status of Selected Essential Trace Elements in Rural South African Women at Delivery. Int. J. Environ. Res. Public. Health.

[27] Duan H., Yu L., Tian F., Zhai Q., Fan L., Chen W. (2020). Gut Microbiota: A Target for Heavy Metal Toxicity and a Probiotic Protective Strategy. Sci. Total Environ..

[28] Assefa S., Köhler G. (2020). Intestinal Microbiome and Metal Toxicity. Curr. Opin. Toxicol..

[29] Tsiaoussis J., Antoniou M.N., Koliarakis I., Mesnage R., Vardavas C.I., Izotov B.N., Psaroulaki A., Tsatsakis A. (2019). Effects of Single and Combined Toxic Exposures on the Gut Microbiome: Current Knowledge and Future Directions. Toxicol. Lett..

[30] Bolan S., Seshadri B., Grainge I., Talley N.J., Naidu R. (2020). Gut Microbes Modulate Bioaccessibility of Lead in Soil. Chemosphere.

[31] Nakamura I., Hosokawa K., Tamura H., Miura T. (1977). Reduced Mercury Excretion with Feces in Germfree Mice after Oral Administration of Methyl Mercury Chloride. Bull. Environ. Contam. Toxicol..

[32] Zhai Q., Qu D., Feng S., Yu Y., Yu L., Tian F., Zhao J., Zhang H., Chen W. (2019). Oral Supplementation of Lead-Intolerant Intestinal Microbes Protects Against Lead (Pb) Toxicity in Mice. Front. Microbiol..

[33] Gupta A., Joia J. (2016). Microbes as Potential Tool for Remediation of Heavy Metals: A Review. J. Microb. Biochem. Technol..

[34] Monachese M., Burton J.P., Reid G. (2012). Bioremediation and Tolerance of Humans to Heavy Metals through Microbial Processes: A Potential Role for Probiotics?. Appl. Environ. Microbiol..

[35] George F., Daniel C., Thomas M., Singer E., Guilbaud A., Tessier F.J., Revol-Junelles A.-M., Borges F., Foligné B. (2018). Occurrence and Dynamism of Lactic Acid Bacteria in Distinct Ecological Niches: A Multifaceted Functional Health Perspective. Front. Microbiol..

[36] Ibrahim F., Halttunen T., Tahvonen R., Salminen S. (2006). Probiotic Bacteria as Potential Detoxification Tools: Assessing Their Heavy Metal Binding Isotherms. Can. J. Microbiol..

[37] Halttunen T., Salminen S., Tahvonen R. (2007). Rapid Removal of Lead and Cadmium from Water by Specific Lactic Acid Bacteria. Int. J. Food Microbiol..

[38] Teemu H., Seppo S., Jussi M., Raija T., Kalle L. (2008). Reversible Surface Binding of Cadmium and Lead by Lactic Acid and Bifidobacteria. Int. J. Food Microbiol..

[39] Bhakta J.N., Ohnishi K., Munekage Y., Iwasaki K., Wei M.Q. (2012). Characterization of Lactic Acid Bacteria-Based Probiotics as Potential Heavy Metal Sorbents. J. Appl. Microbiol..

[40] Kinoshita H., Sohma Y., Ohtake F., Ishida M., Kawai Y., Kitazawa H., Saito T., Kimura K. (2013). Biosorption of Heavy Metals by Lactic Acid Bacteria and Identification of Mercury Binding Protein. Res. Microbiol..

[41] Topcu A., Bulat T. (2010). Removal of Cadmium and Lead from Aqueous Solution by *Enterococcus Faecium* Strains. J. Food Sci..

[42] Halttunen T., Collado M.C., El-Nezami H., Meriluoto J., Salminen S. (2008). Combining Strains of Lactic Acid Bacteria May Reduce Their Toxin and Heavy Metal Removal Efficiency from Aqueous Solution. Lett. Appl. Microbiol..

[43] Daisley B.A., Monachese M., Trinder M., Bisanz J.E., Chmiel J.A., Burton J.P., Reid G. (2019). Immobilization of Cadmium and Lead by *Lactobacillus rhamnosus* GR-1 Mitigates Apical-to-Basolateral Heavy Metal Translocation in a Caco-2 Model of the Intestinal Epithelium. Gut Microbes.

[44] Tian F., Zhai Q., Zhao J., Liu X., Wang G., Zhang H., Zhang H., Chen W. (2012). *Lactobacillus plantarum* CCFM8661 Alleviates Lead Toxicity in Mice. Biol. Trace Elem. Res..

[45] Li B., Jin D., Yu S., Etareri Evivie S., Muhammad Z., Huo G., Liu F. (2017). In Vitro and In Vivo Evaluation of *Lactobacillus delbrueckii* Subsp. *bulgaricus* KLDS1.0207 for the Alleviative Effect on Lead Toxicity. Nutrients.

[46] Zhai Q., Wang G., Zhao J., Liu X., Narbad A., Chen Y.Q., Zhang H., Tian F., Chen W. (2014). Protective Effects of Lactobacillus Plantarum CCFM8610 against Chronic Cadmium Toxicity in Mice Indicate Routes of Protection besides Intestinal Sequestration. Appl. Environ. Microbiol..

[47] Zhai Q., Wang G., Zhao J., Liu X., Tian F., Zhang H., Chen W. (2013). Protective Effects of *Lactobacillus plantarum* CCFM8610 against Acute Cadmium Toxicity in Mice. Appl. Environ. Microbiol..

[48] Yu L., Zhai Q., Yin R., Li P., Tian F., Liu X., Zhao J., Gong J., Zhang H., Chen W. (2017). *Lactobacillus plantarum* CCFM639 Alleviate Trace Element Imbalance-Related Oxidative Stress in Liver and Kidney of Chronic Aluminum Exposure Mice. Biol. Trace Elem. Res..

[49] Yu L., Zhai Q., Liu X., Wang G., Zhang Q., Zhao J., Narbad A., Zhang H., Tian F., Chen W. (2016). *Lactobacillus plantarum* CCFM639 Alleviates Aluminium Toxicity. Appl. Microbiol. Biotechnol..

[50] Bron P.A., Tomita S., Mercenier A., Kleerebezem M. (2013). Cell Surface-Associated Compounds of Probiotic Lactobacilli Sustain the Strain-Specificity Dogma. Curr. Opin. Microbiol..

[51] Kumar N., Kumari V., Ram C., Thakur K., Tomar S.K. (2018). Bio-Prospectus of Cadmium Bioadsorption by Lactic Acid Bacteria to Mitigate Health and Environmental Impacts. Appl. Microbiol. Biotechnol..

[52] Sun Z., Harris H.M.B., McCann A., Guo C., Argimón S., Zhang W., Yang X., Jeffery I.B., Cooney J.C., Kagawa T.F. (2015). Expanding the Biotechnology Potential of Lactobacilli through Comparative Genomics of 213 Strains and Associated Genera. Nat. Commun..

[53] Deutsch S.-M., Mariadassou M., Nicolas P., Parayre S., Le Guellec R., Chuat V., Peton V., Le Maréchal C., Burati J., Loux V. (2017). Identification of Proteins Involved in the Anti-Inflammatory Properties of *Propionibacterium freudenreichii* by Means of a Multi-Strain Study. Sci. Rep..

[54] Ochman H., Selander R.K. (1984). Standard Reference Strains of *Escherichia coli* from Natural Populations. J. Bacteriol..

[55] Rahmouni O., Vignal C., Titécat M., Foligné B., Pariente B., Dubuquoy L., Desreumaux P., Neut C. (2018). High Carriage of Adherent Invasive *E. coli* in Wildlife and Healthy Individuals. Gut Pathog..

[56] Pradel E., Zhang Y., Pujol N., Matsuyama T., Bargmann C.I., Ewbank J.J. (2007). Detection and Avoidance of a Natural Product from the Pathogenic Bacterium *Serratia marcescens* by *Caenorhabditis elegans*. Proc. Natl. Acad. Sci. USA.

[57] Adouard N., Foligné B., Dewulf J., Bouix M., Picque D., Bonnarme P. (2015). In Vitro Characterization of the Digestive Stress Response and Immunomodulatory Properties of Microorganisms Isolated from Smear-Ripened Cheese. Int. J. Food Microbiol..

[58] Malik A.C., Reinbold G.W., Vedamuthu E.R. (1968). An Evaluation of the Taxonomy of *Propionibacterium*. Can. J. Microbiol..

[59] Ameen F.A., Hamdan A.M., El-Naggar M.Y. (2020). Assessment of the Heavy Metal Bioremediation Efficiency of the Novel Marine Lactic Acid Bacterium, *Lactobacillus plantarum* MF042018. Sci. Rep..

[60] Lin D., Ji R., Wang D., Xiao M., Zhao J., Zou J., Li Y., Qin T., Xing B., Chen Y. (2019). The Research Progress in Mechanism and Influence of Biosorption between Lactic Acid Bacteria and Pb(II): A Review. Crit. Rev. Food Sci. Nutr..

[61] Muhammad Z., Ramzan R., Zhang S., Hu H., Hameed A., Bakry A.M., Dong Y., Wang L., Pan S. (2018). Comparative Assessment of the Bioremedial Potentials of Potato Resistant Starch-Based Microencapsulated and Non-Encapsulated *Lactobacillus plantarum* to Alleviate the Effects of Chronic Lead Toxicity. Front. Microbiol..

[62] Mohapatra R.K., Parhi P.K., Pandey S., Bindhani B.K., Thatoi H., Panda C.R. (2019). Active and Passive Biosorption of Pb(II)Using Live and Dead Biomass of Marine Bacterium *Bacillus xiamenensis* PbRPSD202: Kinetics and Isotherm Studies. J. Environ. Manag..

[63] Kirillova A.V., Danilushkina A.A., Irisov D.S., Bruslik N.L., Fakhrullin R.F., Zakharov Y.A., Bukhmin V.S., Yarullina D.R. (2017). Assessment of Resistance and Bioremediation Ability of *Lactobacillus* Strains to Lead and Cadmium. Int. J. Microbiol..

[64] Lin D., Cao H., Zhong Y., Huang Y., Zou J., He Q., Ji R., Qin T., Chen Y., Wang D. (2019). Screening and Identification of Lactic Acid Bacteria from Ya’an Pickle Water to Effectively Remove Pb2. AMB Express.

[65] Foligné B., Breton J., Mater D., Jan G. (2013). Tracking the Microbiome Functionality: Focus on *Propionibacterium* Species. Gut.

[66] Djurasevic S., Jama A., Jasnic N., Vujovic P., Jovanovic M., Mitic-Culafic D., Knezevic-Vukcevic J., Cakic-Milosevic M., Ilijevic K., Djordjevic J. (2017). The Protective Effects of Probiotic Bacteria on Cadmium Toxicity in Rats. J. Med. Food.

[67] Bisanz J.E., Enos M.K., Mwanga J.R., Changalucha J., Burton J.P., Gloor G.B., Reid G. (2014). Randomized Open-Label Pilot Study of the Influence of Probiotics and the Gut Microbiome on Toxic Metal Levels in Tanzanian Pregnant Women and School Children. mBio.

[68] Zhai Q., Wang J., Cen S., Zhao J., Zhang H., Tian F., Chen W. (2019). Modulation of the Gut Microbiota by a Galactooligosaccharide Protects against Heavy Metal Lead Accumulation in Mice. Food Funct..

[69] Bhattacharya S. (2020). The Role of Probiotics in the Amelioration of Cadmium Toxicity. Biol. Trace Elem. Res..

