# Supplementary material for: Assessment of Pb(II), Cd(II), and Al(III) Removal Capacity of Bacteria from Food and Gut Ecological Niches: Insights into Biodiversity to Limit Intestinal Biodisponibility of Toxic Metals
Source: Microorganisms. 2021 Feb 22;9(2):456. doi: 10.3390/microorganisms9020456 (PMC7926695; doi:10.3390/microorganisms9020456)
Supplement: Supplementary file 1 [file microorganisms-09-00456-s001.pdf]

microorganisms

Supplementary file

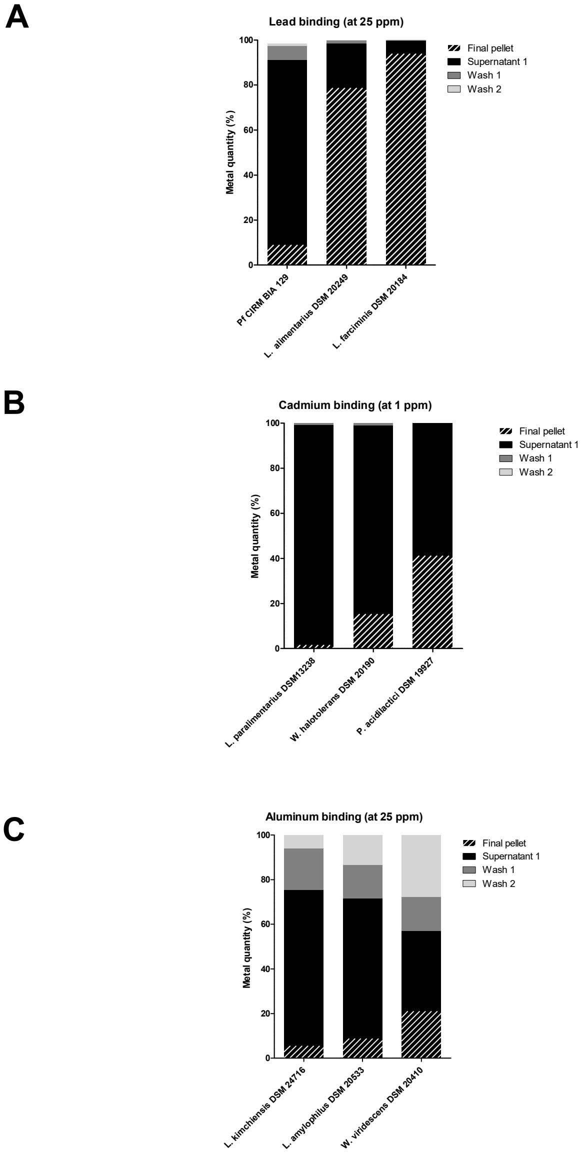

Figure S1: Selected examples of bacterial strains with distinct metal removal capabilities, demonstrating the accuracy of the assays for Pb (A), Cd (B) and Al (C). Metals were quantified in the binding supernatant, in the two washing buffers, and in the final bacterial pellet.

Strain	Cd removal capacity	Cd MIC (ppm)
L. mucosae DSM13345	5.86%	11 ppm
L. ingluviei DSM15946	13.08%	11 ppm
P. inopinatus DSM20285	16.75%	11 ppm
L. hordei DSM19519	10.63%	29 ppm
L. senmaizukei DSM21775	3.54%	29 ppm
L. paralimentarius DSM13238	1.57%	34.5 ppm
L. satsumensis DSM16230	11.39%	34.5 ppm
L. casei DSM20011	23.59%	34.5 ppm
L. gallinarum DSM10532	5.66%	> 55 ppm
W. confusa DSM20196	15.81%	> 55 ppm
L. hominis DSM23910	13.83%	> 55 ppm
L. acidophilus DSM20079	8.06%	> 55 ppm
L. fructosus DSM20349	11.78%	> 55 ppm

Figure S2: Selected examples of bacterial strains with equivalent MICs for Cd but different Cd removal capacities, illustrating the lack of a relationship between metal resistance and metal binding. MICs were determined in 24-hr liquid cultures. The Cd removal capacity is color-coded as weak (0 to 10%: pale green), low (11 to 20%: blue), and moderate (21 to 30%: orange).
